# Genomic Reanalysis of Neurodevelopmental Disorders With and Without Epilepsy: Diagnostic Yield and Mechanisms of Newly Established Diagnoses

**DOI:** 10.3390/genes17091078

**Published:** 2026-09-07

**Authors:** Da Hye Yoon, Chang Ahn Seol, Jung Woo Rhim, Ji Yoon Han

**Affiliations:** 1 Division of Pediatric Neurology, Department of Pediatrics, Severance Children’s Hospital, Yonsei University College of Medicine, Seoul 03722, Republic of Korea; pedydh@yuhs.ac; 2 GC Genome, Yongin 16924, Republic of Korea; changahnseol@gccorp.com; 3 GC Labs, Yongin 16924, Republic of Korea; 4 Department of Pediatrics, College of Medicine, The Catholic University of Korea, Seoul 06591, Republic of Korea; jwrhim@catholic.ac.kr; 5 Department of Pediatrics, Daejeon St. Mary’s Hospital, Daejeon 34943, Republic of Korea

**Keywords:** neurodevelopmental disorders, epilepsy, genomic reanalysis, whole-exome sequencing, diagnostic yield

## Abstract

Background/Objectives: Genomic reanalysis may provide additional molecular diagnoses in patients with neurodevelopmental disorders (NDDs) whose initial genetic testing was nondiagnostic. We evaluated the diagnostic yield and mechanisms of newly established diagnoses in NDDs with and without epilepsy. Methods: We retrospectively reviewed 175 patients with previously unresolved NDDs who underwent whole-exome sequencing between 2015 and 2024. Archived WES variant call format (VCF) files were reannotated using updated databases and variant prioritization criteria, followed by phenotype- and inheritance-informed clinical interpretation. Patients were classified as NDD without epilepsy (*n* = 133) or NDD with epilepsy (NDD+E; *n* = 42). Results: Five new molecular diagnoses were established, yielding an incremental diagnostic yield of 3% (5/175). The yield was numerically higher in the NDD+E group than in the NDD group (7% [3/42] vs. 2% [2/133]), although the difference was not statistically significant (*p* = 0.090). Newly established diagnoses involved *NPRL3*, *RYR1*, *SYNGAP1*, *CSNK2B*, and *SETD5* and arose through distinct mechanisms, including complementary CNV testing with retrospective sequencing review, phenotype- and inheritance-informed reinterpretation, recognition of insufficient initial sequencing coverage, and updated variant interpretation incorporating new evidence. Conclusions: Genomic re-evaluation provided additional diagnoses in a modest but clinically relevant proportion of previously unresolved pediatric NDD cases. Periodic, phenotype-informed reassessment of existing genomic data, together with complementary testing when indicated, may resolve diagnoses missed or uninterpretable at the initial evaluation.

## 1. Introduction

Neurodevelopmental disorders (NDDs) and epilepsy represent a broad spectrum of pediatric neurological conditions characterized by significant clinical and genetic heterogeneity. NDDs, including developmental delay, intellectual disability, and autism spectrum disorder, frequently coexist with epilepsy, with reported comorbidity rates ranging from 20% to 40% depending on the underlying etiology [1,2]. This phenotypic overlap suggests shared neurobiological mechanisms, particularly involving synaptic function, ion channel activity, and transcriptional regulation [3,4,5].

The introduction of next-generation sequencing (NGS), including targeted gene panels and whole-exome sequencing (WES), has substantially improved the diagnostic yield in pediatric neurological disorders [6,7]. Previous studies have reported diagnostic rates of approximately 15–40% in NDD and epilepsy cohorts, depending on patient selection and sequencing strategies [8,9]. Nevertheless, a substantial proportion of patients remain without a definitive molecular diagnosis after initial genetic testing. This may reflect technical limitations of earlier sequencing platforms, incomplete knowledge of gene–disease relationships, evolving understanding of disease mechanisms and inheritance patterns, and changes in variant classification over time [10,11].

Reanalysis of previously generated NGS data provides an opportunity to establish diagnoses without necessarily performing entirely new sequencing. Advances in gene–disease knowledge, updated population and clinical databases, improved bioinformatic approaches, and evolving variant interpretation frameworks may enable previously undetected or inconclusive variants to become diagnostically informative [12,13]. Previous studies have demonstrated additional diagnostic yields of approximately 5–15% after reanalysis, although the magnitude of improvement varies according to the cohort, interval from initial testing, and reanalysis strategy [14,15]. Importantly, new diagnoses may arise through several mechanisms, including recognition of newly established gene–disease associations, reclassification of previously uncertain variants, improved detection of variants missed because of technical limitations, and reassessment of inheritance or phenotype–genotype relationships [16,17].

Although the clinical utility of genomic reanalysis has been increasingly recognized, relatively limited information is available on whether its diagnostic benefit differs between patients with NDD alone and those with coexisting epilepsy. Given the substantial genetic contribution to neurodevelopmental phenotypes accompanied by epilepsy, comparison of these clinically overlapping groups may help identify patient subgroups in whom genomic re-evaluation is particularly informative. Furthermore, examining the mechanisms underlying newly established diagnoses may help identify limitations of previous genetic testing and inform future reanalysis strategies.

In this study, we retrospectively evaluated a pediatric cohort that underwent WES over a 10-year period and re-evaluated previously nondiagnostic cases using updated variant annotation and clinical interpretation. Patients were classified into NDD without epilepsy (NDD group) and NDD with epilepsy (NDD+E group) to compare their clinical characteristics and incremental diagnostic yields following genomic reanalysis. We further characterized the mechanisms underlying newly established molecular diagnoses to evaluate the clinical utility and limitations of genomic reanalysis in a real-world pediatric neurodevelopmental cohort.

## 2. Materials and Methods

### 2.1. Study Population

This retrospective study included patients who underwent WES for neurodevelopmental disorders between 2015 and 2024. During the study period, 375 patients underwent WES testing, of whom 205 remained without a definitive molecular diagnosis after the initial analysis and were considered for reanalysis. Patients were excluded if sufficient clinical information was unavailable, they were lost to follow-up, or consent for reanalysis was not obtained. After applying these exclusion criteria, 175 patients with NDDs were included in the final analysis.

Patients were classified according to the presence of epilepsy into the NDD without epilepsy (NDD group) and NDD with epilepsy (NDD+E group). Demographic and clinical characteristics, including developmental features and severity, neurological and systemic comorbidities, family history, and brain magnetic resonance imaging findings, were retrospectively reviewed from medical records. The severity of DD/ID was classified as mild, moderate, or severe based on available standardized developmental or cognitive assessments, including developmental quotient or intelligence quotient when available, together with documented clinical assessment of developmental and adaptive functioning.

### 2.2. WES Reanalysis and Variant Prioritization

Archived WES variant data were reannotated using an updated in-house bioinformatics pipeline at GC Genome (Yongin, Republic of Korea), based on the GRCh37/hg19 human reference genome. Reanalysis was performed by reannotation of previously generated variant call format (VCF) files rather than by repeat variant calling from raw sequencing or BAM data. Variant annotation incorporated updated gene–disease and variant-level information from clinical and population databases, including OMIM, ClinVar, gnomAD, the Korean Reference Genome Database (KRGDB), HGMD, and ClinGen. gnomAD version 2.1.1 and ClinVar data accessed on 10 May 2026, were used for reanalysis. Transcript-level annotations were based on MANE transcripts where available. Population allele frequencies, predicted molecular consequences, inheritance patterns, and updated clinical classifications were incorporated into variant prioritization.

Candidate variants were prioritized for clinical review if they met at least one of the following criteria: (1) rare high-impact variants, including nonsense, frameshift, and splice-site variants, with a population allele frequency ≤ 0.001; (2) variants classified as pathogenic or likely pathogenic (P/LP) in ClinVar; (3) missense variants with both REVEL and AlphaMissense scores ≥ 0.90; or (4) variants with a SpliceAI score ≥ 0.50. Additional computational and annotation information, including NMD prediction, SIFT, PolyPhen, MutationTaster, CADD, InterVar, and Exomiser results, was available for variant-level review. Because these prioritization criteria were not mutually exclusive, duplicate variants were consolidated before patient-level clinical interpretation.

CNVs were not systematically recalled during the current VCF-based reanalysis. CNV analyses performed as part of the original sequencing workflow had used depth- and coverage-based approaches, including z-score, z-ratio, VisCap, and XHMM, as applicable. In selected cases, complementary CNV testing was performed based on clinical indications. When a clinically relevant CNV was identified by chromosomal microarray analysis (CMA), the corresponding genomic region and previously generated sequencing data were retrospectively reviewed for confirmation and further characterization of the CNV.

### 2.3. Clinical Interpretation and Molecular Diagnosis

Prioritized sequence variants were reviewed according to the ACMG/AMP framework, considering population frequency, inheritance pattern, disease mechanism, gene–disease validity, and phenotype–genotype concordance. For predicted loss-of-function variants, transcript location and the likelihood of nonsense-mediated decay were additionally considered. In silico prediction scores were used for variant prioritization rather than as stand-alone evidence of pathogenicity. CNVs were interpreted according to the classification provided by the clinical laboratory and evaluated for consistency with the patient’s phenotype and expected inheritance pattern.

A new molecular diagnosis was established when a pathogenic or likely pathogenic variant was consistent with the expected inheritance pattern and the patient’s phenotype and had not provided a definitive molecular diagnosis at the time of the original analysis. Variants of uncertain significance, single heterozygous P/LP variants in genes associated exclusively with autosomal recessive disorders, and variants without sufficient phenotype–genotype concordance were not included in the diagnostic yield.

### 2.4. Statistical Analysis

Continuous variables were expressed as mean ± standard deviation or median with interquartile range, as appropriate, and categorical variables as numbers and percentages. Clinical characteristics were compared between the NDD and NDD+E groups using Student’s *t*-test or the Mann–Whitney U test for continuous variables and the chi-square test or Fisher’s exact test for categorical variables, as appropriate. Fisher’s exact test was used when expected cell counts were small.

The incremental diagnostic yield of genomic reanalysis was calculated as the proportion of patients receiving a new molecular diagnosis among the 175 patients included in the reanalysis cohort. Diagnostic yields were calculated separately for the NDD and NDD+E groups and compared using Fisher’s exact test. Given the small number of newly diagnosed cases, the between-group comparison of diagnostic yield was considered exploratory. All statistical tests were two-sided, and a *p* value < 0.05 was considered statistically significant. Statistical analyses were performed using IBM SPSS Statistics for Windows, version 32.0 (IBM Corp., Armonk, NY, USA).

### 2.5. Ethics Statement

This study was conducted in accordance with the principles of the Declaration of Helsinki and was approved by the Institutional Review Board of Daejeon St. Mary’s Hospital, The Catholic University of Korea (IRB No. DC20RASI0079; approval date: 12 March 2025). Written informed consent for genetic reanalysis was obtained from the patients or their legal guardians, as applicable.

## 3. Results

### 3.1. Clinical Characteristics

A total of 175 patients with neurodevelopmental disorders were included in the comparative analysis, comprising 133 patients with NDD without epilepsy and 42 with NDD and epilepsy (NDD+E) (Table 1). Overall, 118 patients (67%) were male. Patients in the NDD+E group were significantly older at diagnosis than those in the NDD group (10.1 ± 5.1 vs. 6.9 ± 7.1 years, *p* = 0.002) and had a longer follow-up duration (4.3 ± 2.0 vs. 2.9 ± 2.1 years, *p* < 0.001).

The frequency of intellectual disability was significantly higher in the NDD+E group than in the NDD group (86% vs. 59%, *p* = 0.001). In contrast, the frequencies of global developmental delay, cognitive delay, language delay, motor delay, social delay, autism spectrum disorder, and neuropsychiatric comorbidities did not differ significantly between the groups. The overall distribution of developmental severity did not differ significantly between the NDD and NDD+E groups (*p* = 0.781). Abnormal brain MRI findings were more frequent in patients with NDD+E than in those with NDD alone (21% vs. 6%, *p* = 0.006). Among specific MRI abnormalities, cerebral atrophy was significantly more common in the NDD+E group (14% vs. 2%, *p* = 0.003), whereas the frequencies of structural malformations and white matter abnormalities did not differ significantly between the groups. No significant between-group differences were observed in adverse perinatal history, neurological or systemic comorbidities, or family history of NDD.

Genomic reanalysis resulted in a new molecular diagnosis in 5 of 175 patients, corresponding to an overall incremental diagnostic yield of 3%. The yield was numerically higher in the NDD+E group than in the NDD group (3/42 [7%] vs. 2/133 [2%]); however, this difference did not reach statistical significance (Fisher’s exact test, *p* = 0.090).

### 3.2. Diagnostic Yield and Newly Established Molecular Diagnoses

Case 1 was an adolescent male with mild intellectual disability (IQ 68) and epilepsy. His mother, maternal grandfather, and maternal uncle also had epilepsy but normal cognitive function, demonstrating a multigenerational pattern consistent with autosomal dominant inheritance. The initial WES analysis had not established a molecular diagnosis. CMA, performed subsequently as part of further clinical genetic evaluation, identified a pathogenic heterozygous deletion at 16p13.3 involving *NPRL3* (approximately 9 kb by CMA). This finding prompted retrospective review of the previously generated WES data, which confirmed the intragenic deletion and more precisely delineated it as an approximately 12 kb heterozygous deletion involving exons 9–15 of *NPRL3*. This established the diagnosis of *NPRL3*-related familial focal epilepsy. The reason for non-detection of this small CNV during the original WES analysis could not be definitively determined retrospectively. This case illustrates a molecular diagnosis achieved through complementary CNV testing followed by targeted reassessment of previously generated sequencing data.

Case 2 was a 5-year-old boy with prominent gross motor delay and mild developmental delay. Initial electromyography and muscle biopsy findings suggested an underlying myopathy; however, the initial genetic evaluation did not establish a molecular diagnosis. His mother and maternal grandfather also had longstanding muscle weakness that did not substantially interfere with routine daily activities but limited exercise and physically demanding activities, suggesting an autosomal dominant pattern of inheritance. Reanalysis identified the heterozygous *RYR1* variant c.14548T>A (p.Tyr4850Asn). Reassessment of the variant in conjunction with the multigenerational muscle phenotype and inheritance pattern supported a diagnosis of an *RYR1*-related disorder. The diagnosis was facilitated by reinterpretation of the variant in the context of the familial phenotype, which had not been sufficiently recognized at the time of the initial analysis.

Case 3 initially presented with language developmental delay and was diagnosed with autism spectrum disorder at 5 years of age. Epilepsy developed at approximately 10 years of age, and antiseizure medication was initiated. Although no further clinical seizures occurred after treatment, epileptiform abnormalities persisted on EEG, requiring continued treatment. Reanalysis identified the pathogenic heterozygous nonsense variant *SYNGAP1* c.3277C>T (p.Gln1093Ter), which had not been detected in the initial analysis because of insufficient sequencing coverage. This finding established the diagnosis of a *SYNGAP1*-related neurodevelopmental disorder.

Case 4 had mild intellectual disability (IQ 60) and attended a mainstream school with additional special education support. From approximately 5 years of age, he experienced recurrent prolonged seizures lasting approximately 5 min during febrile illnesses. At around 8 years of age, seizures also began to occur in the absence of fever, leading to maintenance treatment with antiseizure medication. The heterozygous nonsense variant *CSNK2B* c.139C>T (p.Arg47Ter) had been detected in the original sequencing data but was not considered pathogenic at the time of the initial interpretation. With updated evidence and variant reinterpretation, the variant was subsequently classified as pathogenic, establishing the diagnosis of Poirier–Bienvenu neurodevelopmental syndrome.

Case 5 had global developmental delay beginning at approximately 2 years of age and subsequently required ongoing rehabilitation therapy and special education. The heterozygous *SETD5* c.4271A>G (p.Gln1424Arg) variant was present in the original sequencing data and had initially been considered a variant of uncertain significance, without establishing a molecular diagnosis. During subsequent reassessment, its de novo occurrence was confirmed, and updated variant-level evidence supported reclassification as pathogenic/likely pathogenic. This established the diagnosis of a SETD5-related neurodevelopmental disorder. This case illustrates the diagnostic value of reinterpretation incorporating both updated molecular evidence and inheritance information.

### 3.3. Mechanisms Leading to New Molecular Diagnoses

Genomic reanalysis and complementary genetic evaluation led to five new molecular diagnoses through several distinct mechanisms, including CMA-guided CNV assessment, reassessment of variants in the context of familial inheritance, recognition of technical limitations in the original sequencing data, and updated variant interpretation (Table 2).

In one patient with familial epilepsy, complementary CMA identified a pathogenic heterozygous intragenic deletion involving *NPRL3*, prompting retrospective review of previously generated sequencing data. The deletion was further characterized as involving exons 9–15 and was consistent with the autosomal dominant inheritance of NPRL3-related familial focal epilepsy.

In another patient with a family history of muscle weakness consistent with autosomal dominant inheritance, re-evaluation of an *RYR1* variant, c.14548T>A (p.Tyr4850Asn), together with the familial phenotype and inheritance pattern, led to a diagnosis of an *RYR1*-related disorder. This case illustrates the importance of reassessing variants in the context of updated disease mechanisms and family segregation patterns.

A pathogenic nonsense variant in *SYNGAP1*, c.3277C>T (p.Gln1093Ter), was identified in a patient with neurodevelopmental impairment and epilepsy. Review of the original sequencing data suggested that the corresponding region had not been adequately assessed in the initial analysis because of insufficient sequencing coverage. Reanalysis therefore enabled recognition of a pathogenic variant that had previously escaped detection.

In a fourth patient, the *CSNK2B* nonsense variant c.139C>T (p.Arg47Ter) had been detected in the original sequencing data but had not been considered pathogenic at the time of the initial interpretation. Updated variant-level evidence subsequently supported its classification as pathogenic, establishing a diagnosis of Poirier–Bienvenu neurodevelopmental syndrome.

The heterozygous *SETD5* c.4271A>G (p.Gln1424Arg) variant was present in the original sequencing data but was not considered diagnostic at the time of the initial analysis. During subsequent reassessment, its de novo occurrence was confirmed, and updated variant-level evidence supported its classification as pathogenic/likely pathogenic. This established the diagnosis of a SETD5-related neurodevelopmental disorder. This case illustrates the diagnostic value of reinterpretation incorporating both updated molecular evidence and inheritance information.

Overall, the newly established diagnoses were attributable not to a single mechanism but to complementary effects of updated variant interpretation, phenotype- and inheritance-informed reassessment, recognition of technical limitations, and additional genetic testing. These findings highlight the potential clinical value of periodic genomic re-evaluation of previously nondiagnostic cases, even when the overall incremental diagnostic yield is modest.

## 4. Discussion

In this retrospective study, genomic re-evaluation of 175 patients with previously unresolved NDDs established five new molecular diagnoses, corresponding to an incremental diagnostic yield of 3%. Although the overall yield was modest, the newly established diagnoses arose through distinct mechanisms, including complementary CNV testing followed by retrospective review of sequencing data, reassessment of a variant in the context of familial inheritance and phenotype, recognition of a pathogenic variant affected by insufficient initial sequencing coverage, and updated variant interpretation incorporating newly available evidence. The diagnostic yield was numerically higher in patients with NDD and epilepsy than in those with NDD alone (7% vs. 2%), although the difference did not reach statistical significance. These findings illustrate that re-evaluation of previously nondiagnostic genomic data can remain clinically informative even when the incremental diagnostic yield is relatively low.

The incremental diagnostic yield observed in our study was lower than that reported in many previous reanalysis studies. Previous studies have reported additional diagnostic yields exceeding 10% in selected NDD cohorts, although substantial variation exists according to patient selection, sequencing strategy, reanalysis interval, and analytic approach [18]. For example, reanalysis of WES data after 12 months increased the diagnostic yield by approximately 11%, with new diagnoses attributable not only to advances in gene–disease knowledge but also to updated phenotypic information and improvements in bioinformatic analysis [19]. However, a recent longitudinal study involving routine biannual exome reanalysis reported a diagnostic rate of only 2.6% among initially nondiagnostic cases, with reanalysis yields ranging from 0% to 7.3% across phenotypic subgroups [20,21], which is comparable to the 3% observed in our cohort. Differences in reanalysis yield likely reflect heterogeneity in the initial sequencing strategy, duration between initial testing and reanalysis, availability of parental samples, incorporation of complementary testing, evolution of clinical phenotypes, and criteria used to define a molecular diagnosis. In addition, our stringent definition required a P/LP variant with an appropriate inheritance pattern and phenotype–genotype concordance, while VUSs and isolated carrier findings were excluded from the diagnostic yield. These factors may partly account for the relatively modest yield observed in our study.

Despite the modest overall yield, the higher proportion of new diagnoses in the NDD+E group is noteworthy. Three of 42 patients with NDD+E (7%) received a new molecular diagnosis compared with two of 133 patients with NDD alone (2%). Although this approximately five-fold numerical difference did not reach statistical significance (*p* = 0.090), likely owing to the small number of newly diagnosed cases, it is consistent with the substantial contribution of monogenic disorders to combined neurodevelopmental and epileptic phenotypes. Exome sequencing has previously demonstrated high diagnostic yields in individuals with NDD accompanied by additional neurological manifestations [22]. More recently, a large meta-analysis found that the diagnostic yield of WES was higher in NDD patients with co-occurring epilepsy than in the overall NDD population (35.6% vs. 35.3%), although the magnitude of this difference was small [18,19]. Thus, our findings cannot establish epilepsy as an independent predictor of successful reanalysis, but they raise the possibility that patients with combined NDD and epilepsy may represent an informative subgroup for periodic genomic re-evaluation. Larger cohorts are required to determine whether this numerical difference represents true enrichment.

The five newly diagnosed cases also demonstrate that successful genomic re-evaluation may involve more than automated reannotation of existing sequence variants. In the patient with familial epilepsy, complementary CMA identified a pathogenic heterozygous intragenic deletion involving *NPRL3*, which prompted retrospective review of previously generated sequencing data and further characterization of the deletion. Loss-of-function variants, including intragenic deletions, are an established molecular mechanism of *NPRL3*-related epilepsy, which typically follows autosomal dominant inheritance and exhibits considerable phenotypic variability [23]. The occurrence of epilepsy in the proband, his mother, maternal grandfather, and maternal uncle, despite normal cognition in the affected relatives, illustrates intrafamilial variability in neurodevelopmental manifestations. Importantly, this case should not be interpreted as a CNV newly detected by VCF-based reanalysis; rather, it demonstrates the complementary role of CNV testing and retrospective reassessment of previously generated sequencing data in unresolved cases.

The *RYR1* case illustrates a different challenge: interpretation of a rare variant in the context of phenotype and inheritance. The proband presented with prominent gross motor delay and myopathic findings, while his mother and maternal grandfather had longstanding muscle weakness, suggesting autosomal dominant transmission. *RYR1*-related disorders encompass a broad clinical and genetic spectrum, including both dominant and recessive congenital myopathies, malignant hyperthermia susceptibility, exertional rhabdomyolysis, and overlapping phenotypes [24,25]. Consequently, interpretation of rare *RYR1* missense variants can be challenging when molecular findings are considered independently of segregation and detailed neuromuscular phenotyping. In our patient, integration of the multigenerational phenotype and inheritance pattern during reassessment contributed to recognition of an *RYR1*-related disorder. This case emphasizes that genomic re-evaluation should integrate updated clinical and familial information rather than rely solely on computational reannotation. Importantly, establishing an RYR1-related diagnosis may have direct clinical implications, including counseling regarding malignant hyperthermia susceptibility, perioperative precautions, and assessment of potentially affected family members.

The *SYNGAP1* case demonstrates the persistent relevance of technical limitations in earlier NGS testing. The patient initially presented with language delay and autism spectrum disorder and subsequently developed epilepsy, a phenotype compatible with the established spectrum of *SYNGAP1*-related neurodevelopmental disorder, in which developmental impairment, autism spectrum features, and epilepsy frequently coexist [26]. The pathogenic nonsense variant c.3277C>T (p.Gln1093Ter) had not been identified during the initial analysis, with retrospective review suggesting insufficient sequencing coverage of the corresponding region. Although sequencing technologies and bioinformatic pipelines have improved substantially, inadequate coverage remains a potential cause of false-negative results. This case underscores the importance of considering technical limitations of the original assay when the clinical phenotype remains strongly suggestive of a monogenic disorder.

The *CSNK2B* and *SETD5* cases highlight the dynamic nature of variant interpretation. The *CSNK2B* nonsense variant c.139C>T (p.Arg47Ter) had been present in the original sequencing data but was not considered diagnostic at the time of initial interpretation. Subsequent accumulation of evidence supported its pathogenicity and established the diagnosis of Poirier–Bienvenu neurodevelopmental syndrome. *CSNK2B*-related disease is now recognized as a neurodevelopmental disorder with variable intellectual disability and epilepsy, with pathogenic variants spanning multiple variant classes [27]. Similarly, the de novo *SETD5* c.4271A>G (p.Gln1424Arg) variant was not considered diagnostic in the initial evaluation but was subsequently recognized as clinically significant after incorporation of updated variant-level and segregation evidence. De novo pathogenic variants in *SETD5* are an established cause of intellectual disability and developmental disorders [28]. These cases illustrate how variants that are nondiagnostic at one point in time may become clinically interpretable as gene–disease knowledge, population databases, segregation information, and classification frameworks evolve.

Taken together, the mechanisms underlying the five new diagnoses indicate that genomic re-evaluation should be viewed as a dynamic process rather than simply automated reannotation against updated databases. In the present study, the formal WES reanalysis consisted primarily of reannotation of previously generated VCF files using updated databases and prioritization tools, without repeat variant calling from raw sequencing data. Nevertheless, clinically meaningful diagnoses also emerged through integration of complementary testing, reassessment of familial inheritance, recognition of technical limitations in the original sequencing data, and incorporation of updated variant-level evidence. Recent exome reanalysis studies similarly distinguish variant-level reevaluation from case-level reevaluation and demonstrate that both contribute substantially to newly established molecular diagnoses [18,19,20]. Our findings therefore support a phenotype-informed approach in which existing genomic data are periodically revisited together with longitudinal clinical and familial information and, when indicated, complementary genetic testing.

From a clinical perspective, establishing a molecular diagnosis after an initially nondiagnostic evaluation may have implications beyond diagnostic closure. A revised diagnosis can refine genetic counseling and recurrence-risk assessment, inform surveillance for disorder-specific complications, guide management in selected conditions, and enable appropriate testing of family members. It may also provide patients and caregivers with an explanation for the underlying disorder and reduce the need for repeated diagnostic investigations. However, genomic reanalysis is not yet implemented uniformly in routine clinical practice, and the optimal timing and scope of re-evaluation remain uncertain. Our findings support incorporating periodic, phenotype-informed genomic reassessment into the longitudinal care of selected patients with unresolved NDDs, particularly when the phenotype evolves, new family information becomes available, or the original testing had technical or interpretive limitations.

This study has several limitations. First, it was a retrospective, single-center study with only five newly diagnosed cases, resulting in limited statistical power, particularly for comparison of diagnostic yields between the NDD and NDD+E groups. The higher yield observed in the NDD+E group should therefore be regarded as exploratory. Second, patients underwent genetic testing over a 10-year period, during which sequencing platforms, coverage, annotation databases, and variant interpretation practices evolved. Although this heterogeneity reflects real-world clinical practice and provides a rationale for re-evaluation, it may also have influenced the likelihood and mechanisms of subsequent diagnosis. Third, the current WES reanalysis was based on reannotation of previously generated VCF files rather than repeat variant calling from raw sequencing or BAM data. Therefore, variants absent from the original VCF because of inadequate coverage, calling limitations, structural variation, low-level mosaicism, or other technical factors could not be systematically recovered through reannotation alone. CNVs were also not systematically recalled during the current reanalysis. Fourth, complementary testing contributed to the diagnosis in selected cases, particularly the NPRL3 case, and therefore not all five diagnoses can be attributed solely to VCF-based WES reanalysis. Finally, detailed information regarding the precise basis for historical variant classification was not available for all cases, particularly when subsequent testing or reinterpretation had been performed at another institution.

In conclusion, genomic re-evaluation of previously unresolved NDD cases provided an additional molecular diagnosis in 3% of patients in this real-world pediatric cohort. Although the incremental yield was modest, it was comparable to that reported in a recent routine exome reanalysis study, and the newly established diagnoses arose through diverse mechanisms extending beyond database reannotation alone. Patients with coexisting epilepsy showed a numerically higher diagnostic yield than those with NDD alone, although this difference was not statistically significant. Our findings support periodic, phenotype-informed re-evaluation of unresolved genomic findings and highlight the complementary roles of updated variant interpretation, longitudinal clinical information, familial segregation, assessment of technical limitations, and additional genetic testing in resolving previously nondiagnostic cases.

## 5. Conclusions

Genomic re-evaluation of previously unresolved NDD cases provided an additional molecular diagnosis in 3% of patients in this real-world pediatric cohort. Although the incremental yield was modest, the newly established diagnoses arose through diverse mechanisms extending beyond database reannotation alone. Patients with coexisting epilepsy showed a numerically higher diagnostic yield than those with NDD alone, although this difference was not statistically significant. Periodic, phenotype-informed genomic re-evaluation, together with complementary genetic testing when indicated, may help resolve previously nondiagnostic cases.

## Figures and Tables

**Table 1:** Clinical characteristics of the study population.

	Total (*n* = 175)	NDD (*n* = 133)	NDD with Epilepsy (*n* = 42)	*p*-Value
Sex (male, %)	118 (67%)	91 (68%)	27 (64%)	0.706
Age at diagnosis	7.7 ± 6.9	6.9 ± 7.1	10.1 ± 5.1	0.002
Follow-up duration	3.3 ± 2.1	2.9 ± 2.1	4.3 ± 2.0	<0.001
**Adverse perinatal history**	16 (9%)	12 (9%)	4 (9%)	1.000
Preterm birth	9 (5%)	6 (5%)	3 (7%)	0.450
Low birth weight	9 (5%)	6 (5%)	3 (7%)	0.450
NICU admission history	14 (8%)	11 (8%)	3 (7%)	1.000
**Neurodevelopmental characteristics ^†^**				
Global developmental delay	107 (61%)	85 (64%)	22 (52%)	0.206
Cognitive delay	104 (59%)	83 (62%)	21 (50%)	0.207
Language delay	100 (57%)	79 (59%)	21 (50%)	0.290
Motor delay	16 (9%)	13 (10%)	3 (7%)	0.765
Social delay	70 (40%)	55 (41%)	15 (36%)	0.590
Intellectual disability	115 (66%)	79 (59%)	36 (86%)	0.001
Autism spectrum disorder	49 (28%)	34 (26%)	15 (36%)	0.238
Neuropsychiatric comorbidities	7 (4%)	5 (4%)	2 (5%)	0.674
Regression	0	0	0	
**Severity of DD/ID**				0.781
Mild	93 (53%)	72 (54%)	21 (50%)	0.724
Moderate	43 (25%)	33 (25%)	10 (24%)	1.000
severe	39 (22%)	28 (21%)	11 (26%)	0.526
**Neurological and systemic comorbidities**				0.836
Hypotonia	8 (4%)	5 (4%)	3 (7%)	0.400
Spasticity	4 (2%)	3 (2%)	1 (2%)	1.000
Ataxia	2 (1%)	1 (1%)	1 (2%)	0.423
Movement disorder	1 (1%)	1 (1%)	0 (0%)	1.000
Microcephaly	1 (1%)	1 (1%)	0 (0%)	1.000
Macrocephaly	1 (1%)	1 (1%)	0 (0%)	1.000
Dysmorphic features	10 (6%)	8 (6%)	2 (5%)	1.000
Visual impairments	1 (1%)	1 (1%)	0 (0%)	1.000
Hearing impairment	5 (3%)	4 (3%)	1 (2%)	1.000
Obesity	7 (4%)	7 (5%)	0 (0%)	0.991
Others	17 (10%)	13 (10%)	4 (10%)	1.000
brain MRI				0.006
normal	158 (90%)	125 (94%)	33 (79%)	
abnormal	17 (10%)	8 (6%)	9 (21%)	
structural malformation	4 (2%)	2 (2%)	2 (5%)	0.244
white matter abnormality	8 (5%)	5 (4%)	3 (7%)	0.400
cerebral atrophy	8 (5%)	2 (1%)	6 (14%)	0.003
others	1 (1%)	0 (0%)	1 (2%)	0.240
Family history of NDD	23 (13%)	19 (14%)	4 (10%)	0.601
Family history of epilepsy	8 (5%)	4 (3%)	4 (10%)	0.095
Yield of reanalysis of NGS	5 (3%)	2 (2%)	3 (7%)	0.090

^†^ Neurodevelopmental characteristics were non-mutually exclusive; therefore, percentages do not sum to 100%. NDD, neurodevelopmental disorder; NICU, neonatal intensive care unit; DD, developmental delay; ID, intellectual disability; MRI, magnetic resonance imaging.

**Table 2:** Newly established molecular diagnoses identified through genomic reanalysis and reinterpretation.

Case	Clinical Phenotype	Gene	Variant	Inheritance	Initial Interpretation	Final Classification (ACMG/AMP Criteria)	Mechanism Leading to Molecular Diagnosis	Molecular Diagnosis
1	familial focal epilepsy with mild intellectual disability	*NPRL3*	Heterozygous deletion involving exons 9–15	AD	No molecular diagnosis established by initial WES	P (PVS1, PM2, PP4 ^†^)	Complementary CMA detection followed by retrospective WES confirmation and refinement	NPRL3-related familial focal epilepsy
2	Muscle weakness; family history of muscle weakness	*RYR1*	c.14548T>A (p.Tyr4850Asn)	AD	VUS	P/LP (PM2, PP3, PP4 ^‡^)	Phenotype- and inheritance-informed reinterpretation	RYR1-related disorder
3	Autism spectrum disorder with epilepsy	*SYNGAP1*	c.3277C>T (p.Gln1093Ter)	AD	Not detected	P (PVS1, PM2)	Insufficient coverage in the initial sequencing data	SYNGAP1-related intellectual developmental disorder
4	Intellectual disability with epilepsy	*CSNK2B*	c.139C>T (p.Arg47Ter)	AD	Updated variant interpretation	P (PVS1, PM2, PP4 ^§^)	Updated variant interpretation	Poirier–Bienvenu neurodevelopmental syndrome
5	Global developmental delay	*SETD5*	c.4271A>G (p.Gln1424Arg)	AD	Present in original data; not considered diagnostic	P/LP (PS2, PM2, PP3, PP4)	Variant reclassification incorporating de novo and updated evidence	SETD5-related neurodevelopmental disorder

^†^ NPRL3 CNV interpretation was additionally considered according to ACMG/ClinGen recommendations for copy-number variants. PP1 may be applicable if familial segregation of the deletion was molecularly confirmed. ^‡^ PP1 may be applicable if segregation of the RYR1 variant was molecularly confirmed in affected family members. ^§^ PS2 may be applicable if the CSNK2B variant was confirmed to have occurred de novo. AD, autosomal dominant; CMA, chromosomal microarray analysis; P, pathogenic; LP, likely pathogenic; VUS, variant of uncertain significance; WES, whole exome sequencing.

## Data Availability

The data supporting the findings of this study are available from the corresponding author upon reasonable request. The data are not publicly available due to privacy and ethical restrictions related to patient genetic and clinical information.
